# Expanding our thought horizons in systems biology and medicine

**DOI:** 10.3389/fsysb.2024.1385458

**Published:** 2024-03-06

**Authors:** Jennifer C. Lovejoy

**Affiliations:** ^1^ Phenome Health, Seattle, WA, United States; ^2^ Institute for Systems Biology, Seattle, WA, United States; ^3^ Center for Phenomic Health, The Buck Institute for Research on Aging, Novato, CA, United States

**Keywords:** systems biology, multi-omic analyses, behavioral science, environmental science, precision medicine, artificial intelligence, exposome

## Introduction

In 2021, the founding editor of Frontiers in Systems Biology wrote about the need for integration in systems biology, and the irony of the growing fragmentation of both disciplines and toolsets that has begun to characterize the field, much as it did reductionist biology (Vodovotz, 2021). He also wrote of the need to change mindsets to address this tendency toward fragmentation, which is often the best starting place for discussing the challenges that face systems biology and systems medicine today.

## Mindsets and methods

Behavioral scientists define mindset as a set of assumptions, methods, or notions held by a person or a group, often unconsciously. We may have held a set of beliefs for so long, or find them so culturally embedded, that we do not recognize the mindset we have. On the positive side, so-called growth mindsets have been associated with educational attainment and improved mental health (Dweck and Yeager, 2020) On the negative side, “implicit bias” - negative, unconscious biases regarding race, socioeconomic status, mental health, or physical characteristics–is sadly common among the public and health professionals (Meidert et al., 2023).

As scientists, we are trained in critical thinking, hypothesis testing, and other approaches to assure that our experimental designs and data interpretations are objective and reasonably free from bias. Because of this, we may assume that mindsets do not affect our thinking and our research. Unfortunately, however, there are many examples of bias in science–from deliberately omitting contradictory papers or data that do not fit with our hypotheses to casting results in a specific light out of the best intentions (Cope and Allison, 2010). Because of the unconscious nature of mindsets, our scientific biases are most inadvertent.

## The importance of the exposome

One common and likely unrecognized bias in systems biology and medicine research is failing to account for the impact of cognitive, behavioral, and environmental factors–i.e., the “exposome”–on the biology of the organism being studied.

There are many examples of the bidirectional interaction of biology with cognitive and behavioral factors: inflammation with stress and depression (Felger, 2018; Vodovotz et al., 2024), sex hormones with appetite and mood (Gorczyca et al., 2016), chronic stress with abdominal fat deposition (Delker et al., 2021), and many more.

Furthermore, it is widely recognized that lifestyle behaviors such as diet, physical activity, and sleep have a significant impact on physiology, health, and aging (Vodovotz et al., 2020; Zhang et al., 2023). In some cases, genetic factors play a role in determining one’s response to lifestyle change and may be an unrecognized cause for variability in outcomes (Zubair et al., 2019).

Considerations of the effects of environment on biology include factors typically thought of as “environmental”, such as temperature, humidity, air pollution, and noise level (Gao et al., 2022), as well as the larger environmental context, such as the regulatory and policy environment. For human biology, the environment includes the systems in which we live and work such as healthcare, government, and culture (including views of race, gender, social class, and physical ability). These factors have a major influence on health and physiology, either directly (e.g., lack of equitable access to healthcare) or through effects on cognitive and emotional factors (Riley, 2012; Paradies et al., 2015; Ding et al., 2022; Abrue et al., 2024).

While consistently accounting for behavioral and environmental factors in clinical studies should seem obvious, it is also important in research on animal models. For example, mice are nocturnal animals, but studies with mice are typically conducted during the day, a mouse’s inactive or sleep period. Research has shown that there are measurable differences in mice tested during their inactive vs. active periods (i.e., day vs. night) (Tsao et al., 2022) which may influence interpretation of results.

Another environmental factor influencing animal studies is how the animals are housed. Group- or pair-housed mice (Nagy et al., 2002) and monkeys (Charbonneau et al., 2022) have different physiological and behavioral characteristics than do individually housed animals. Furthermore, an animal’s sex, genetic background, and position in the social hierarchy may influence their response to the housing environment.

While behavioral and environmental factors are not always controllable, the real problem is when studies do not measure or report exposome data. Despite cogent arguments for the importance of including the exposome in multi-omic studies (Logan et al., 2018; Price et al., 2022), many studies in systems biology and medicine fail to measure and account for behavioral and environmental factors, which can lead to incorrect interpretation of experimental results.

## Challenges for systems biology and systems medicine

The work of our group and others suggests that to optimally implement systems biology and medicine research, it is important to longitudinally measure as many variables about the system as possible to inform new insights and generate new hypotheses (Yurkovich et al., 2023). There are, however, challenges to this approach.

Simply gathering the breadth and depth of data for systems studies can be difficult. It is not uncommon to see “omics” studies focusing on a single data type–e.g., genomics, proteomics, or metabolomics–rather than multiple measures that explore the genome and phenome simultaneously at a deep level. And, as discussed above, cognitive, behavioral, and environmental data are often omitted. Lastly, many studies omit valuable longitudinal data collection or fail to consider the dynamic nature of physiological systems, including circadian, seasonal, and menstrual/estrous cycle variations, which can be essential to understanding biological data (Boyce et al., 2020; Shen et al., 2024).

There are likely several reasons for these gaps. Below I have listed some of the challenges with collecting broad multi-omic data and posed some specific questions for researchers to consider.

**1) Phenomic assays are expensive.** While costs of whole genome sequencing have declined dramatically since the Human Genome Project, costs of phenomic assays have not followed suit. In particular, the costs of clinical laboratory assays, which are essential for systems medicine research, are prohibitive. However, other omics measures also present a cost barrier in both human and animal research, as can electronic systems to capture behavioral data in animals.

Questions for the research community:

• *What new and emerging technologies can lower the cost of gathering a breadth of phenomic data?*
• *How can alternative, lower-cost methods for existing clinical assays be validated so they will be accepted by both the research and clinical community?*

**2) Collecting broad phenomic markers can involve significant participant burden.** In human studies, simply collecting enough blood to run multiple omics and clinical assays can be difficult and, in groups like infants or the elderly, potentially impossible. Gut microbiome tests require stool collection and compliance is often low in clinical trials. Cognitive and behavioral testing can also be burdensome, particularly if relying on extensive questionnaires or in-person neuropsychological interviews.

Questions for the research community:

• *What new and emerging technologies will allow for accurate measurement of multiple biomarkers in small amounts of blood or other biological fluid? How can these novel approaches be validated for clinical use?*
• *What innovative technologies will allow for easier, accurate collection of gut microbiome data?*
• *What novel approaches, including digital methods and artificial intelligence (AI), can be leveraged for cognitive testing, both in neurodegenerative disease and in healthy individuals?*

**3) Phenomic and exposome measures can be imprecise.** The validity and robustness of research-grade phenomic assessments can be variable and, in some cases, technology is still nascent. Wearable devices are important for measuring the exposome and are being more widely used in systems biology research (Babu et al., 2024). Some lifestyle factors, such as physical activity and sleep (Evenson et al., 2015; Svensson et al., 2019), as well as heart rate variability for stress (Jerath et al., 2023), can be assessed from wearable devices. Dietary intake is more challenging to accurately represent in clinical studies. While there have been some technological advances, e.g., using digital technologies for analyzing photos of food, researchers typically rely on dietary questionnaires, which can be inaccurate and also pose significant research burden (Neve et al., 2022). Similarly, psychological and psychosocial assessments typically rely on questionnaires. While validated questionnaires have good reliability and reproducibility in the populations for which they were originally developed, they often perform less well in diverse populations (Stewart et al., 2012).

Questions for the research community:

• *What are the best technologies for accurately quantifying behavioral variables such as activity, sleep, diet, and stress?*
• *How can researchers best assure the validity of psychological and psychosocial assessments in diverse populations?*
• *What are the best technologies for accurately quantifying the micro-environment in relation to physiology or health?*

**4) Limited cross-disciplinary implementation of analytical tools**. While arguably a challenge in all domains of science, this challenge is ironically greater in systems biology which nearly always involves cross-disciplinary approaches. Staying abreast of new technologies for analytical measurement of biomarkers and environmental factors, as well as advances in data science and statistical methods, requires diligence and ongoing training on the part of researchers. Often, development of computational tools necessarily lags development of new experimental tools but advances in computational and analytic methodology is essential. Data interoperability is also a major consideration. Lastly, given the rapid developments in the field of artificial intelligence (AI), significant consideration should be given to the role of AI - especially generative AI - in systems biology and medicine.

Questions for the research community:

• *How is cross-disciplinary training on tools and methodologies best accomplished?*
• *What can academic institutions, science policymakers, and other stakeholders in science do to encourage cross-disciplinary use of analytical tools?*
• *What are the opportunities and challenges for AI in systems medicine and systems biology? How do we ensure adequate understanding of the limitations of AI in research and clinical care?*

**5) Significant regulatory, educational, and process barriers to implementing systems approaches in healthcare**. It has been estimated that there is a time lag of anywhere from 5 to 25 years for clinical research results to be translated into use in a healthcare setting (Morris et al., 2011). With systems medicine, we are not only talking about translating research to practice but also implementing a paradigm shift in how we think about human health. Achieving this shift will require changing many current processes, providing education and training, and, in some cases, changing regulations (e.g., updating the U.S. Genetic Information Non-discrimination Act of 2008 to include provisions around life insurance and long-term care).

Questions for the research community:

• *What can we learn from different disciplines that have succeeded in major paradigm shifts about how to approach this challenge?*
• *What are the key policies and regulations that impact the implementation (or lack of implementation) of systems approaches in healthcare? Are there model systems in certain countries that make implementing systems medicine easier?*
• *How can we narrow the time gap between scientific discoveries and innovations in systems biology and the translation to clinical care?*

## Conclusion

Biomedicine is at a pivotal time where our widely bemoaned “broken healthcare systems” are ripe for change as more stakeholders realize that the reductionist and siloed approaches to biological and clinical research are no longer effective. Although there is substantial work to be done, this is truly an exciting time for everyone working in systems biology and systems medicine. Famed systems thinker Donella Meadows (Meadows, 2012) describes “expanding thought horizons” this way:

“Seeing systems whole requires more than being “interdisciplinary,” if that word means, as it usually does, putting together people from different disciplines and letting them talk past each other. Interdisciplinary communication works only if there is a real problem to be solved, and if the representatives from the various disciplines are more committed to solving the problem than to being academically correct. They will have to go into learning mode, to admit ignorance and be willing to be taught, by each other and by the system. It can be done. It’s very exciting when it happens.”
